# Femoral derotational osteotomy level does not effect resulting torsion

**DOI:** 10.1186/s40634-020-00227-9

**Published:** 2020-03-04

**Authors:** Eric W. Edmonds, Corey B. Fuller, Megan E. Jeffords, Christine L. Farnsworth, Amelia M. Lindgren, Andrew T. Pennock, Vidyadhar V. Upasani

**Affiliations:** 1 Department of Orthopaedic Surgery, University of California, San Diego, USA; 2 Division of Orthopedics, Rady Children’s Hospital, 3020 Children’s Way, MC 5062, San Diego, CA 92123 USA

**Keywords:** Femoral Anteversion, Femoral Anteversion correction technique, Femoral osteotomy, Distal, proximal, femoral version measurement

## Abstract

**Purpose:**

The purpose of this study was to assess the effect on femoral torsion by rotational osteotomies at three different levels as measured in 3D using both the mechanical and the anatomic axes.

**Methods:**

Ten cadaveric lower extremities underwent femoral osteotomies perpendicular to the anatomic axis (AA) at three levels: subtrochanteric, mid-diaphyseal and supracondylar. Parallel pins were placed, one in each femur segment. Computed tomography (CT) was acquired in post-osteotomies neutral position, then post-external rotation of the femur at each osteotomy level. Femurs were returned to neutral rotation between imaging exams. Using 3D CT reconstructions, custom software calculated femoral torsion (angle between the femoral neck axis and the posterior condylar axis in the transverse plane) and pin angle between segments, reoriented to both the mechanical axis (MA) and the AA. Pin angle and torsion change were compared for the three osteotomy locations (regression analysis and ANOVA performed).

**Results:**

Two specimens were omitted (inadequate imaging); the remaining eight donors were 55–90 years old (mean: 64 ± 15 years), CT confirmed no bony defects. All three levels of osteotomy demonstrated significant correlations between the amount of rotation at the osteotomy (pin angle change) and the resulting change in femoral torsion (R square range 0.658–0.847). No significant differences were found between osteotomy level in torsion (MA:*p* = 0.285, AA:*p* = 0.156) or in pin angle (MA:*p* = 0.756, AA:*p* = 0.753).

**Conclusions:**

Performing a corrective rotational osteotomy orthogonal to the AA achieves the desired effect on MA regardless of location. This suggests that a surgeon’s osteotomy level choice may be based on other risks/benefits of the various techniques.

## Background

Derotational osteotomies to correct pathologic femoral antetorsion in children with neuromuscular disease continue to be commonplace, with a growing body of literature to support utilization in adolescent or adult patients with patellofemoral issues or extra-articular femoroacetabular impingement [1–4]. Pathologic femoral torsion can be treated surgically with derotational osteotomies at the subtrochanteric level (proximal), in the mid-diaphysis (midshaft), or just above the diaphyseal-metaphyseal junction (distal). The appropriate location for the osteotomies, with their various associated risks and benefits, is debated in the literature [5–10]. The ability to achieve the desired amount of rotation has not been previously compared between the three different locations.

There is some concern that the osteotomies can affect more than just the desired rotation [7]; therefore, understanding the effect on measurable rotation is important for each of these locations. All locations of osteotomy appear to potentially affect the frontal plane with proximal osteotomies resulting in potential varus deformation and distal osteotomies resulting in potential valgus deformation [11]. Therefore, the question regarding the ability for each osteotomy location to achieve only the desired effect is important. Past study has only compared each level of osteotomy against itself, by using different imaging modalities in the comparison (x-ray compared to 2D CT, for example) [12]. Yet, no previous study has directly compared the ability of each level of osteotomy to produce the desired or expected rotation against an osteotomy at a different level on the same bone. Nor has there been previous study to evaluate the potential deformity in 3D.

Although the treating surgeon is clinically concerned with the mechanical axis, the surgical osteotomies are made orthogonal, or perpendicular, to the anatomic axis. The normal anatomy of the femur is slightly curved; therefore, there is a potential discrepancy between intended and actual changes to the mechanical and anatomic axes when performing corrective femoral osteotomy.

## Purpose

The purpose of this study was to assess the effect on torsion by rotational osteotomies at three different levels in the femur as measured in 3D using both the mechanical and the anatomic axes. We hypothesized that rotational osteotomies performed orthogonal to the anatomic axis would have increasing ability to predictably change the mechanical axis from proximal to distal cuts due to the convergence of the two axes at the distal femur.

## Methods

Cadaveric lower extremities had femoral osteotomies at clinically useful proximal, mid-shaft and distal locations. External rotation was performed at each level, measured by pin rotation between bone segments. CT images were reconstructed in 3D and custom software used to semi-automatically compute actual amount of rotation induced (pin angle change) and femoral torsion following rotation at each level.

### Specimens and preparation

Ten fresh-frozen adult cadaveric lower extremities (hemi-pelvis to foot) were acquired as power analysis determined that *n* = 9 would be needed in order to determine population differences in femoral torsion (data used from Kaiser et al. [12]). Specimens with no hip, knee or ankle arthritis, osteoporosis, prior extremity or joint infection, prior extremity surgery, previous femur, tibia or fibular fracture or other known systemic musculoskeletal disorder affecting bone or soft tissue anatomy were requested. Initially, each lower extremity underwent torsional profile CT scanning, following our institutional standard clinical technique of scanning the hip, knee and ankle (0.625 mm thick cuts, GE LightSpeed VCT 64-Slice, Piscataway, NJ, USA). CT images were viewed to confirm lack of joint and bone pathologies.

Lower extremities were then defrosted for 48 h at room temperature. The entire femur was exposed with a lateral incision, and the femoral length was measured from the greater trochanter to the most distal point between the femoral condyles. A mark was made in each femur indicating half of its length. Femoral osteotomies were performed perpendicular to the anatomic axis at all three levels: proximal (just distal to the lesser trochanter), midshaft (at the half-length mark) and distal (in the supracondylar region). Each osteotomy site was fixed into the native anatomic alignment (0 degrees of rotation) using a plate (4.5 mm narrow stainless steel locking compression plate, 7 holes, Synthes, West Chester, PA, USA) held in place with 2 or 3 self-tapping cortical screws (stainless steel 5.0 mm locking and 4.5 mm non-locking × 38-46 mm, Synthes, West Chester, PA, USA) on either side of the osteotomy location.

### Surgical rotation technique and measurement

**Fig. 1:**
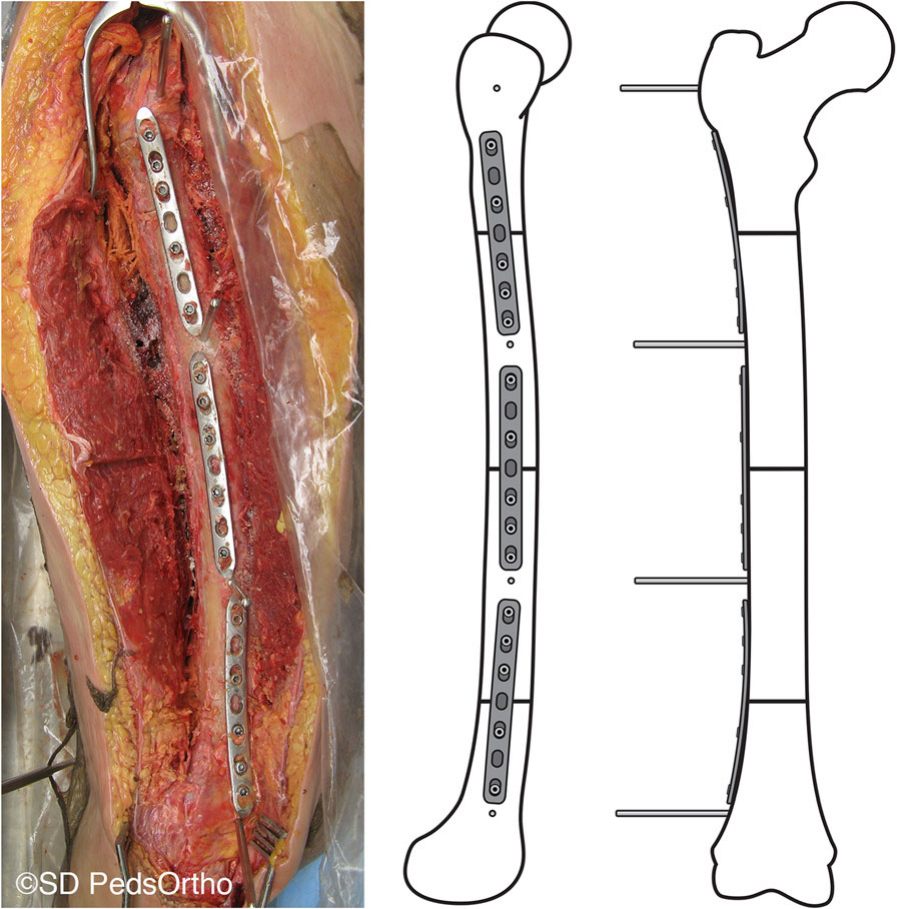
Photo (lateral view) and schematic representation (lateral and anteroposterior views) of osteotomy level locations with and plate and screw placement

**Fig. 2:**
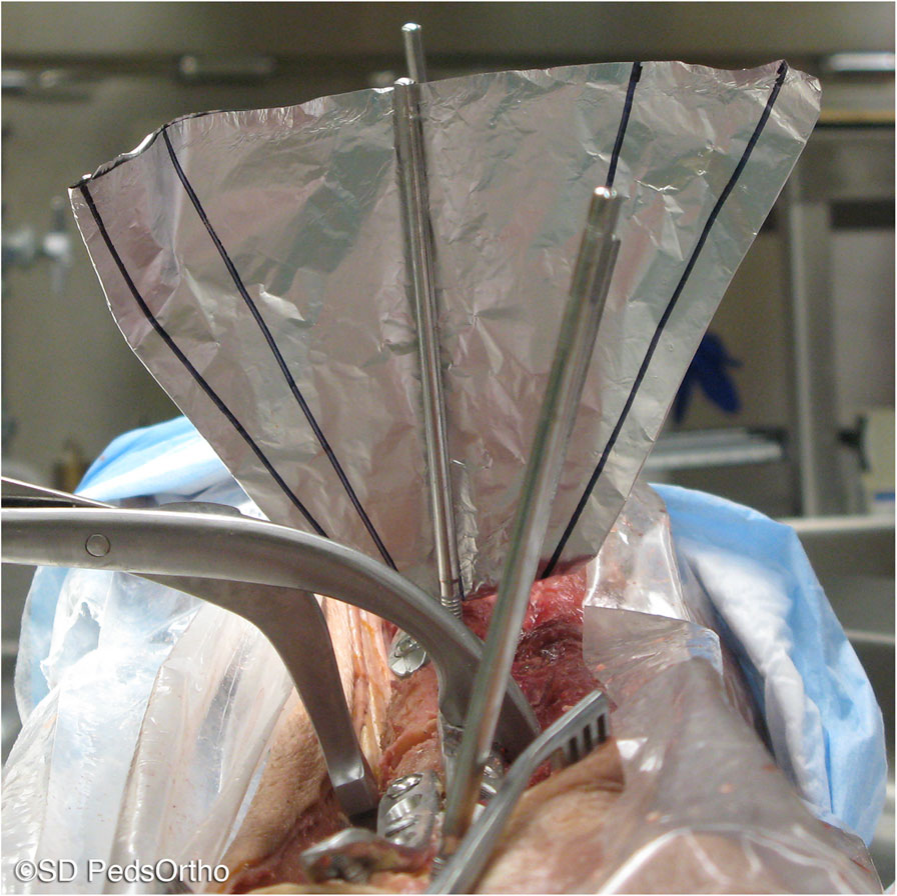
External rotation through a middle level femoral osteotomy measured by pins placed in adjacent segments of the osteotomy. The more distal segment is rotated externally by 20 degrees, and then held in place with a plate and locking screws

Four 5.0 mm self-drilling Schanz screws (pins, DePuy Synthes, New Brunswick, NJ, USA) were placed parallel to each other into each femur, one pin in each of the four bone segments created by the osteotomies (Fig. 1). One of the plates was removed and, mimicking rotational correction techniques used in the operating room, the more distal portion of the femur was rotated externally approximately 15 to 20 degrees (clinically applicable amounts of rotation) as viewed along the long axis of the femur, aligning two adjacent pins to a goniometer. Cortex screw holes were made in order to secure the osteotomy site with a plate and screws. The rotation was returned to neutral, and this was repeated for the remaining two other osteotomy levels. CT scans were then performed of the entire femur in a lateral position with the knee flexed 30 degrees as measured using a goniometer placed along the length of the femur and tibia. An initial CT scan was completed with plates securing all osteotomy levels in the neutral position. Then the femur was rotated externally through one osteotomy level, fixed in place with plates and screws using pre-drilled holes (Fig. 2), CT scan was repeated, then the rotation at that level was returned to the neutral position, prior to rotation at the next level. This was repeated at each osteotomy level with the sequence of first, second and third level to be rotated systematically alternated between proximal, midshaft and distal levels to remove effects of previous rotations.

### Femoral torsion measurement technique

Mimics software (v.19, Materialise, Leuven, Belgium) was used to generate true 3D reconstructions of the proximal and distal ends of each femur from CT DICOM images. The pins placed into each of the four bone segments were also modeled using Mimics. All 3D reconstructions were exported as stereolithography (STL) files. Femoral heads were then separated from the proximal femur reconstruction using 3-matic Medical (Materialise NV, Leuven, Belgium). All 3D models were imported into custom MATLAB (Mathworks, Natick, MA, USA) software for femoral torsion and pin angle calculations. Femoral torsion was automatically calculated using a custom a MATLAB program, thus eliminating human measurement error [13]. Femur models were reoriented based on the mechanical axis (MA) and the anatomical axis (AA). MA was between the center of the femoral head best fit sphere and midpoint between the distal femoral condyles. AA was a center line along the shaft of the femur, drawn between points 10 cm distal to the head and 10 cm proximal to the condyles.

**Fig. 3:**
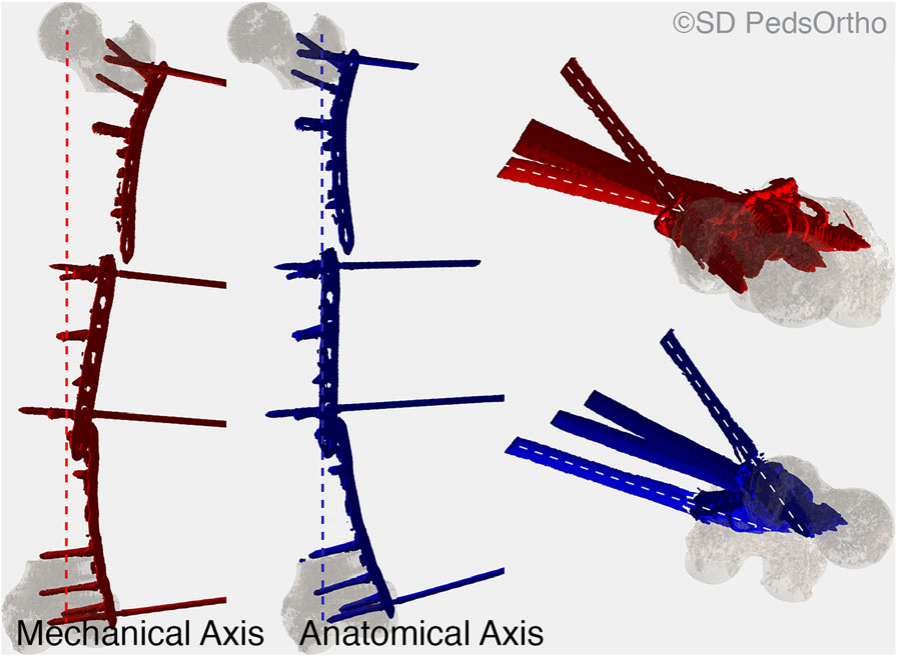
3D reconstructions of proximal and distal femur segments from one of the specimens with plate and screw reconstructions incorporated (red shows mechanical axis alignment from the center of the femoral head to a mid-point between the femoral condyles, blue shows anatomical axis along the femoral shaft length). Dashed lines on axial views on the right side of the figure show Pin Angle, automatically calculated in the mechanical and the anatomical axes

Femoral torsion was calculated as the angle between the femoral neck axis and the posterior condylar axis in the transverse plane. Torsion was set positive when the femoral neck was anteverted (in front of the posterior condylar axis) and negative when the femoral neck was retroverted (behind the posterior condylar axis) to represent clinical understanding of femoral torsion. Pin position was also determined in the MA and AA transverse planes by identifying centerlines along each of the screws from centroid positions. Pin angle was calculated using the angle change between the most proximal and distal pins (Fig. 3). External rotation of the pins was set positive.

For each osteotomy site, the change in femoral torsion and change in pin angle were calculated and compared between osteotomy levels (proximal, midshaft and distal) by the computer modeling and the intended amount of rotation during the surgery. The difference in angle calculated from 3D CT reconstructions between adjacent pins in each osteotomy site was considered variance related to technique rather than affect due to location of osteotomy.

### Statistical analysis

Basic descriptive statistics are presented. The Shapiro-Wilk test of normality and Levene’s test of homogeneity of variance was performed on all continuous data prior to analysis. All data was found to be normally distributed. Analysis of variance was used to evaluate differences in torsion between osteotomy locations. Simple linear regression analysis was used to evaluate the effect of pin angle change on torsion. All statistical analysis was performed using SPSS (version 12; SPSS, Chicago, IL, USA). Statistical significance was defined as *p* < 0.05.

## Results

Two of the specimens were excluded from the study due to inadequate imaging wherein the computer could not identify the appropriate landmarks to create the 3D renderings. The resulting eight lower extremities were from three female and five male donors, between the ages of 55 and 90 years (mean: 64 ± 15 years). The initial CT scans confirmed that each was without any defect in the bone or joints. Following proximal, midshaft and distal osteotomies, but prior to rotation, femoral torsion mean using the MA was 12.0 ± 21.4° (range 39.7° [anteversion] to − 21.5° [retroversion]). Femoral torsion using the AA was 14.6 ± 21.8° (range 43.0° [anteversion] to − 19.0° [retroversion]).

**Fig. 4:**
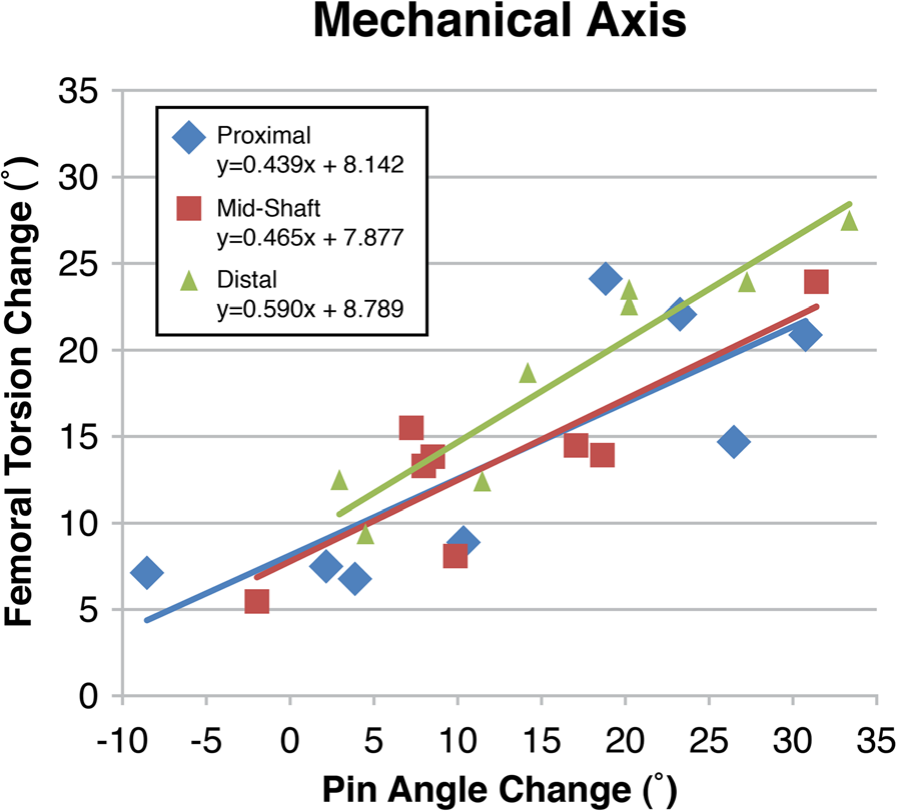
Change in femoral torsion in the mechanical axis with pin angle, rotation through the osteotomy, change. All correlations are significant (p.0.05)

Using both the MA and the AA, all three levels of femoral osteotomy demonstrated significant correlations between the amount of rotation at the osteotomy (pin angle change) and the resulting change in femoral torsion (Table 1). In relation to the MA (Fig. 4), following proximal rotation, the mean pin angle change was 13.4 ± 13.5° (range:-8 ° to 31°) and the mean femoral torsion change was 14.0 ± 7.4° (range: 24° to 7°; β = 0.439). Midshaft rotation changed the mean pin angle by 12.4 ± 9.9° (range:-2° to 31°) and the mean femoral torsion by 13.8 ± 5.4° (range: 24° to 6°; β = 0.466). Distal rotation changed the mean pin angle by 16.5 ± 10.6° (range: 3° to 33°) and the mean femoral torsion by 18.5 ± 6.7° (27° to 9°; β = 0.590).

**Table 1:** Effect of femoral external rotational osteotomy location on femoral torsion correction (negative pin angle change is external rotation)

		Pin angle Change (°)	Femoral Torsion Change (°)	Regression Analysis		
		Range	Range	R square	β	Significance
Mechanical Axis	Proximal	−8.5 – 30.8	24.1–6.8	0.654	0.439	0.015
	Midshaft	−2.0 – 31.4	24.0–5.5	0.724	0.466	0.0007
	Distal	3.4–33.4	27.4–9.3	0.892	0.590	< 0.001
Anatomic Axis	Proximal	−4.0 – 27.8	24.1–6.0	0.641	0.532	0.017
	Midshaft	−5.9 – 31.7	22.4–3.4	0.770	0.463	0.004
	Distal	0.4–32.4	28.0–8.2	0.862	0.520	0.001

**Fig. 5:**
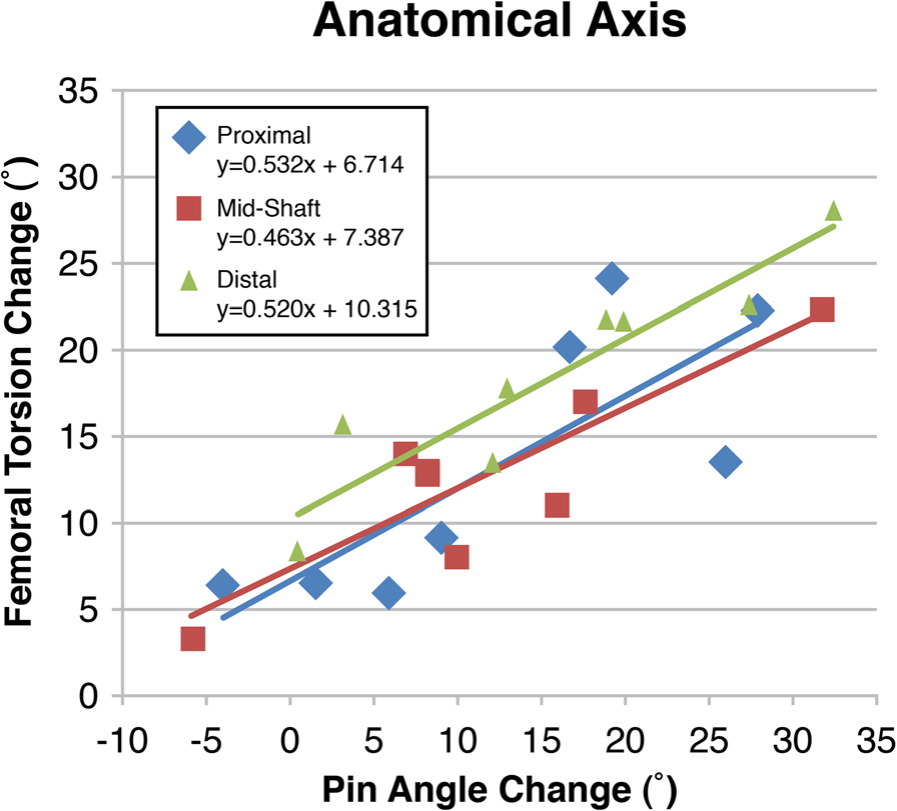
Change in femoral torsion in the anatomical axis with pin angle, rotation through the osteotomy, change. All correlations are significant (p.0.05)

Similarly, when calculated based on the AA (Fig. 5), proximal rotation resulted in a mean pin angle change of 12.9 ± 11.5° (range:-4° to 28°) and a mean femoral torsion change of 13.5 ± 7.6° (range: 24° to 6°; β =0.532). Midshaft rotation changed the mean pin angle by 11.6 ± 10.9° (range:-6° to 32°) and the mean femoral torsion by 12.6 ± 5.7° (range: 22° to 3°; β =0.462). Distal rotation resulted in a mean pin angle change of 15.8 ± 11.0° (range: 0° to 32°) and a mean femoral torsion change of 18.8 ± 6.3° (28° to 8°; β =0.520). No significant differences were found in torsion change based on osteotomy level (MA: *p* = 0.285, AA: *p* = 0.156) or pin angle (MA: *p* = 0.756, AA: *p* = 0.753) in relation to either the MA or AA. In both MA and AA, each degree of rotation change resulted in 3D torsion change of 0.44 to 0.59 degree (Figs. 4 and 5).

The difference between 3D CT reconstructions between adjacent pins in each osteotomy site related to intended rotation versus rotation obtained with plating fixation technique was calculated to be 5.2° in the MA and 5.6° in the AA. This is considered the variability amongst the specimens due to the derotational technique, not due to the level of the osteotomy.

## Discussion

Previous research evaluating osteotomies to affect pathologic femoral antetorsion have tended to either assess the final clinical outcome or compare the ability to measure change in rotation between imaging modalities, rather than between the level of osteotomy [1–4, 12, 14–16]. The risk inherent in this surgery is that osteotomies of the femur performed orthogonal to the anatomic axis may not fully achieve the desired mechanical axis changes, and the location of the corrective rotation may dampen those results. Yet, the technology to accurately assess the true amount of rotation achievable by femoral osteotomy versus the anticipated amount based on perceived surgical intervention was not previously available. The present study was able to utilize custom software and fine cut CT images to create 3D renderings and make these measurements without introducing subjective measurement error, thereby comparing outcomes of rotation directly related to the level of the osteotomy on both mechanical and anatomic axes. Despite concerns, neither the level of the osteotomy nor the osteotomies being orthogonal to the anatomic axis appear to under correct the desired change in mechanical axis during surgery.

There is anecdotal evidence, or at least a common perception amongst surgeons that the amount of correction performed during femoral derotation procedures is reduced once the patient has recovered. The common belief is that the muscle memory or residual proximal weakness of the hip external rotators causes the patient to revert back to their old way of walking because that feels “more normal” to them. This study was performed to disprove this widely held belief, but our hypothesis was instead disproven. Despite the apparent differences between anatomic axis and mechanical axis and the differential separation of those vectors proximal to distal, our study demonstrated that the rotation in pin placement nearly mirrored the observed femoral rotation. However, the intended rotation during the procedure was on average 5 degrees more than the computer measured observed rotation.

Regardless of the level that the femoral osteotomy was performed (proximal, midshaft, or distal) the effect on the torsion was similar: external rotation resulted in decreased femoral antetorsion and with similar error of measurable rotation. It is important to understand that β does not refer to correlation, but rather the slope of the correlation. More specifically, no matter the level of the osteotomy, and no matter whether the computer analyzed the femur in line with the mechanical or the anatomic axis, the regression analysis suggests that about 1 degree of rotation in the osteotomy can achieve about 0.5 degrees of change in the 3D femoral torsion. There are no previous studies with which to compare these findings, and it is therefore important to note that perceived rotation did not align with actual rotation on a 1 to 1 scale.

Our results suggest that the clinical observation that inspired this experiment of observed reduction in intended rotation during recovery was not related to technical error of cutting orthogonal to the anatomic axis with the intent of rotating the mechanical axis, but that the dampened result was still occurring – even in the cadaver. Further evaluation of our results, suggests that the effect may be related to fixation of the osteotomy. During the procedures, it was noted that when non-locking plates and screws were utilized during testing that there was often motion noted (despite the rigid fixation) when the osteotomies were fixed in position after rotation. This has since been noted clinically, as well. That pins placed to measure the desired rotation will lose some of their rotation after the plate is seated against the femur with screw fixation. Perhaps this is a natural phenomenon, in that the cuts of the osteotomy want to rotate to restore its original position, or perhaps it is unperceived ridges along the bone force under-correction as the plate seats up against the cortex. Either way, the concern about losing some of the corrective rotation appears to be validated, but the etiology of this problem remains unanswered.

There are a few limitations to this study that need to be considered. First, is that there was a relatively small sample size. Two of the specimens were not included because the obtained CT images did not include enough of the femur for the custom software to identify landmarks to make the measurements. Although, the measurements of rotation could have been made by hand, we did not want to introduce subjective human measurement error. Second, we did not directly measure the anterior femoral bow of the femur. There is evidence that the anterior femoral bow of the femur affects femoral anteversion measurements and neglecting it can falsely increase femoral anteversion measurements [17]. However, this likely applies more to 2D proximal femur measurement techniques of anteversion. This study uses 3D CT data of the entire length of the femur, which takes into account anterior femoral bow and theoretically avoids this error. Third, despite utilizing customized software to measure true anteversion, we are still unable to fully define the functional axis for both the proximal and distal aspects of the femur. But, by using the machine processing, we eliminated human error for each femur, and guaranteed that each would be measured utilizing the same landmarks across all femurs. Finally, this is not a clinical assessment of each of these osteotomy levels, and there is no gait analysis or reduction in patellofemoral pain to be analyzed. But, the clinical significance of this work cannot be refuted, as the results tender the ability for a surgeon to make decisions regarding the level of the osteotomy based on comfort level, fixation type, and other reasons of importance rather than one level being more predictable of success than another.

## Conclusion

The discussion of appropriate level of osteotomy to treat pathologic femoral torsion can now move beyond the argument regarding which level is most accurate concerning an anticipated change in torsion and the true change. Although, the issues concerning shifts in varus or valgus were not studied here, these may continue to guide treatment choice with the knowledge that there is no difference in the achievable rotation. Future studies from our lab will focus on the changes in the frontal plane with axial plane correction, plus fixation techniques. Moreover, the effect on both mechanical axis and anatomic axis appears to be similar even though the osteotomies are made orthogonal to only the anatomic axis. Further clinical study can now be conducted in a comparison with full equipoise regarding the accuracy of the various osteotomy levels to achieve a desired rotation.

## Data Availability

The datasets used and/or analyzed during the current study are available from the corresponding author on reasonable request.
